# Hydralazine loaded nanodroplets combined with ultrasound-targeted microbubble destruction to induce pyroptosis for tumor treatment

**DOI:** 10.1186/s12951-024-02453-0

**Published:** 2024-04-20

**Authors:** Shuting Huang, Mengmeng Shang, Lu Guo, Xiao Sun, Shan Xiao, Dandan Shi, Dong Meng, Yading Zhao, Xiaoxuan Wang, Rui Liu, Jie Li

**Affiliations:** 1 Department of Ultrasound, Qilu Hospital of Shandong University, Jinan, Shandong 250012 China; 2 Department of Ultrasound, Qilu Hospital (Qingdao) of Shandong University, Qingdao, Shandong 266035 China

**Keywords:** Ultrasound-targeted microbubble destruction, Pyroptosis, GSDME, Hydralazine, Nanodroplet

## Abstract

Pyroptosis, a novel type of programmed cell death (PCD), which provides a feasible therapeutic option for the treatment of tumors. However, due to the hypermethylation of the promoter, the critical protein Gasdermin E (GSDME) is lacking in the majority of cancer cells, which cannot start the pyroptosis process and leads to dissatisfactory therapeutic effects. Additionally, the quick clearance, systemic side effects, and low concentration at the tumor site of conventional pyroptosis reagents restrict their use in clinical cancer therapy. Here, we described a combination therapy that induces tumor cell pyroptosis via the use of ultrasound-targeted microbubble destruction (UTMD) in combination with DNA demethylation. The combined application of UTMD and hydralazine-loaded nanodroplets (HYD-NDs) can lead to the rapid release of HYD (a demethylation drug), which can cause the up-regulation of GSDME expression, and produce reactive oxygen species (ROS) by UTMD to cleave up-regulated GSDME, thereby inducing pyroptosis. HYD-NDs combined with ultrasound (US) group had the strongest tumor inhibition effect, and the tumor inhibition rate was 87.15% (HYD-NDs group: 51.41 ± 3.61%, NDs + US group: 32.73%±7.72%), indicating that the strategy had a more significant synergistic anti-tumor effect. In addition, as a new drug delivery carrier, HYD-NDs have great biosafety, tumor targeting, and ultrasound imaging performance. According to the results, the combined therapy reasonably regulated the process of tumor cell pyroptosis, which offered a new strategy for optimizing the therapy of GSDME-silenced solid tumors.

**Supplementary Information:**

The online version contains supplementary material available at 10.1186/s12951-024-02453-0.

## Introduction

Cancer is among the greatest human health issues globally. The World Health Organization predicts that by 2030, the number of cancer deaths will increase by up to 80% [1]. Therefore, more cell death modalities need to be explored to effectively treat cancer. The discovery of new forms of programmed cell death (PCD) and their actions within tumoral genesis has contributed to an update of antitumor therapeutic strategies [2, 3].

Pyroptosis, a new characteristic form of PCD, was firstly identified in Shigella fowleri infected macrophages in 1992 [4]. Its physical features included the swelling of organelles and the rupture of cell membranes, which released pro-inflammatory cytokines and cell contents [5]. Earlier studies identified Gasdermin D (GSDMD), a pyroptosis executor, which is cleaved in immunocytes following activation by caspase-1 and caspase-11/4/5 [6, 7]. Recently, Gasdermin E (GSDME), another member of the pyroptosis family, was found to trigger pyroptosis in a variety of cancer cells [8–11]. Unlike GSDMD, activated caspase-3 cleaved GSDME, producing GSDME-N fragments that form pores within membranes, resulting in pyroptosis [12]. However, in the majority of cancer cells, including those from the stomach, breast, and colon, GSDME is silenced as a result of promoter DNA hypermethylation [13]. Therefore, specifically inducing pyroptosis by upregulating GSDME expression in tumor cells may be a promising antitumor strategy.

Hydralazine (HYD), a conventional drug used to treat hypertension and heart failure, can reduce the level and activity of DNA methyltransferase1 (DNMT1) by inhibiting mitogen-activated protein kinase, thus having the property of DNA demethylation [14–16]. However, there are certain drawbacks to using HYD alone, such as quick clearance, systemic side effects, and low concentration at the site of the tumor. Furthermore, it was considered that HYD monotherapy may only upregulate GSDME expression in tumors and not induce pyroptosis [17]. Cleavage of GSDME is usually caused by chemotherapeutic drugs, but drug resistance and severely adverse effects limit its application in biomedicine [18–20]. Therefore, there is an urgent need to find a novel, collaborative method to maximize the induction of pyroptosis to achieve the therapeutic effect of cancer.

The tumor microenvironment (TME) has obvious physiological features, including low pH, up-regulation of enzyme expression and hypoxia, so TME stimulus-responsive nanoparticles can specifically release drugs in tumor sites, thereby effectively targeting tumors and increasing therapeutic efficacy [21–23]. Recently, multifunctional nanodroplets, a nanoscale ultrasound contrast agents (UCAs) with both diagnostic and visualization therapeutics, have been exploited [24, 25]. With ultrasound irradiation, the liquid-core nanodroplets were transformed to gaseous-core microbubbles to enhance ultrasound imaging contrast, a transformation referred to as acoustic droplet vaporization (ADV) [26–28]. Meanwhile, the sonoporation effect resulting from ultrasound-targeted microbubble destruction (UTMD) increases the permeability of local microvessels and cell membranes, thus increasing intake rate [29]. In addition, under the UTMD effect, the microbubbles formed by nanodroplet phase transition undergo expansion and contraction causing cavitation effect, which releases reactive oxygen species (ROS) [30]. ROS was found to be a crucial factor in GSDME cleavage [31–34], but the application of UTMD in pyroptosis has not been reported.

**Fig. 1:**
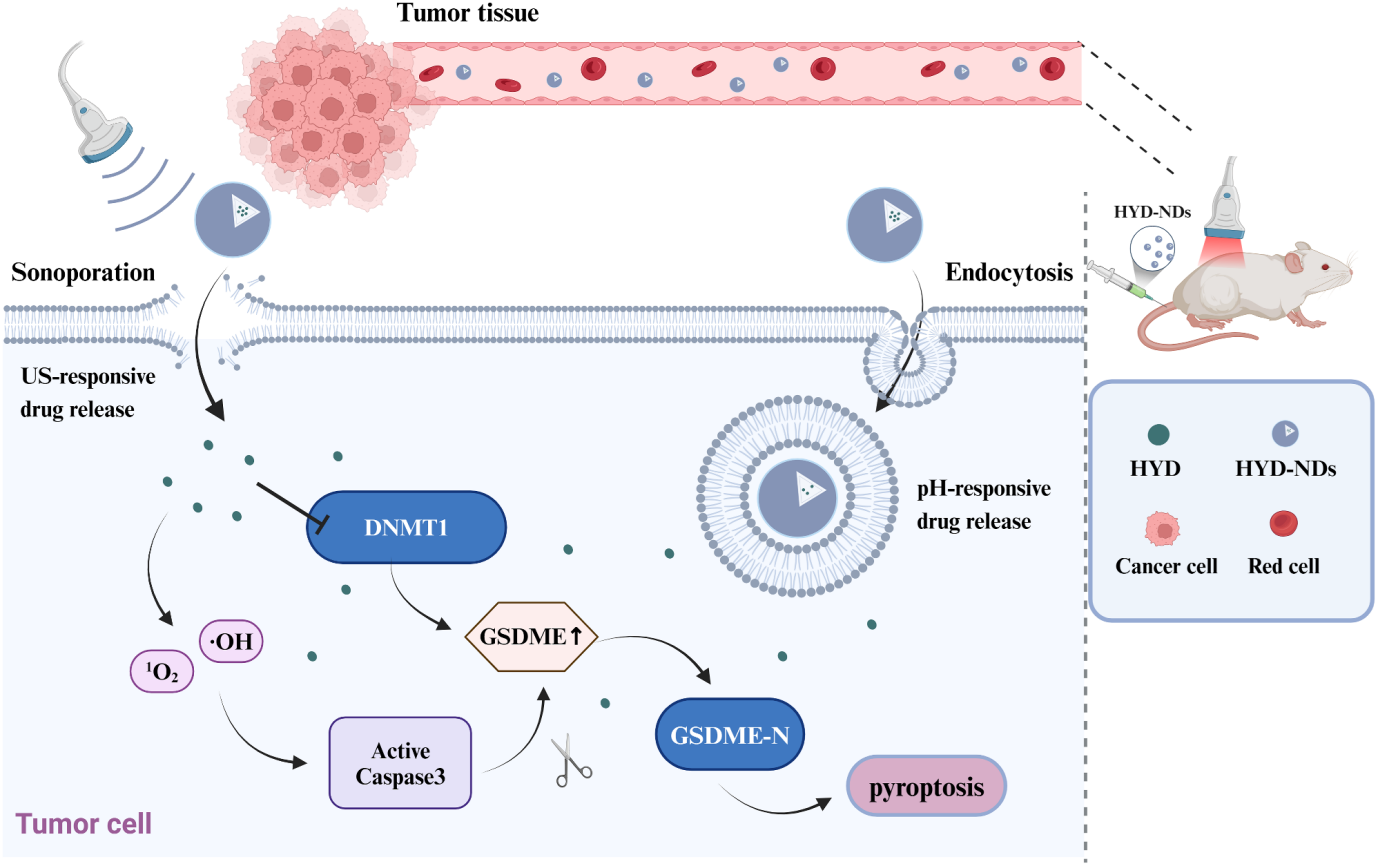
Schematic diagram of the antitumor effect of HYD-NDs in combination with UTMD

In this study, the hydralazine-loaded nanodroplets (HYD-NDs) that were given dual pH and ultrasound (US) responsiveness, in combination with UTMD was recruited to program pyroptosis for the treatment of solid tumors while minimizing systemic toxicity. HYD-NDs is a nano-drug carrier designed with perfluorohexane (PFH) as core and O-carboxymethyl chitosan (O-CMC) as coating material. Thanks to its pH responsiveness, this nanodroplet can accumulate at the tumor site. Then, low-dose ultrasound irradiation of the tumor site induces ROS generation, penetrates the cell membrane to activate caspase-3, and releases HYD to increase the expression of GSDME, a pyroptosis substrate specifically for ROS mediated caspase-3 cleavage, which synergistically leads to the pyroptosis of cancer cells (Fig. 1). HYD-NDs can be used as a good biocompatible platform for inducing pyroptosis, and its combination with UTMD provides a new strategy for optimizing the treatment of solid tumors with GSDME silencing.

## Materials and methods

### Materials

Hydralazine was acquired from GlpBio (Shanghai, China). Lactate dehydrogenase (LDH) Release Assay Kit and Reactive Oxygen Species Assay Kit were bought from Beyotime (Shanghai, China). N-acetyl-Lcysteine (NAC), a ROS inhibitor was bought from Macklin (Shanghai, China). Anti-DFNA5/GSDME was bought from Abcam (Cambridge, UK) and Proteintech (Wuhan, China). Cleaved Caspase-3 (Cleaved-CASP3) antibody was acquired from Cell Signaling Technology (Danvers, USA).

### Cell culture

The American Type Culture Collection provided 4T1 (mouse breast cancer) cells. The cell lines were cultivated at 37 °C in a humid environment with 5% CO2 in RPMI 1640 medium (BasalMedia, Shanghai, China) supplied with 10% fetal bovine serum (FBS, Gibco, Carlsbad, USA) and 1% penicillin-streptomycin (Solarbio, Beijing, China). The study’s cell lines were verified through the use of short tandem repeats (STRs) profiling.

### Animal model

The Qilu Hospital of Shandong University’s Laboratory Animal Ethics Committee authorized the protocols for the animal experiments. Female BALB/c mice (4–5 weeks) were purchased from charles river (Zhejiang, China). A suspension of 4T1 cells (1 × 10^6^ cells) was subcutaneously injected into each mouse’s right flank to create the tumor model.

### Preparation of (HYD-)NDs

Both NDs and HYD-NDs were produced through emulsion homogenization. Lecithin, PFH and Tween were added to deionized water and treated with an ultrasonic crusher (UP250, Scientz, China) at 150 w under ice bath conditions for 5 min (alternating between working for 10 s and resting for 10 s). After that, O-CMC solutions with or without HYD were added drop by drop while continuing the ultrasonic treatment for 5 min. Next, mixture was centrifuged at 300 rpm for 5 min. After being collected, the intermediate layer was centrifuged for 15 min at 12,500 rpm. The resulting precipitate was washed three times using PBS as purified NDs or HYD-NDs. For later use, purified NDs and HYD-NDs were re-suspended in PBS.

### Characterization of (HYD-)NDs

The size distribution and zeta potential of HYD-NDs were examined by dynamic light scattering (DLS, Malvern Zetasizer Nano, UK). HYD-NDs were analyzed using transmission electron microscope (TEM, Tokyo, Japan) to confirm their morphological and dispersive properties. The stability of HYD-NDs was assessed from tracking the change in size at predetermined intervals within 50% FBS over a 48-hour period.

The ultraviolet absorption spectra of HYD-NDs, NDs, and HYD were obtained using an ultraviolet-visible spectrophotometer (DeNovix, Wilmington, USA). The standard curves of HYD drawn, and HYD’s loading efficiency (LE) and entrapment efficiency (EE) in HYD-NDs were calculated. Through dialysis at 37 °C, the drug release curve of HYD-NDs was established. A 2 mL solution of HYD-NDs was put in a dialysis bag and cultured in PBS buffers with various pH values on a shaker at 37 °C and 100 rpm in order to test the pH responsiveness of HYD-NDs. Then 1 mL aliquots were taken out and repeated with equal volumes of PBS at predetermined intervals. We measured the amount of HYD released with an ultraviolet spectrophotometer. To assess its ultrasonic sensitivity, HYD-NDs was enclosed within a dialysis bag, with or without exposure to ultrasonic irradiation, followed by the aforementioned treatment steps to generate an ultrasonic response release profile. A WED-300 focused ultrasound therapeutic instrument (Shenzhen, China) was applied, with the following main parameters: transducer size (irradiation area) of 6.0 cm^2^, working frequency of 1.0 MHz, intensity of 1.0 W/cm^2^, and irradiation time of 30 s.

### In vitro cytotoxicity

The cytotoxicity to 4T1 cells was assessed via Counting Kit-8 (CCK-8) assay. The microplate reader (Infinite M200, TECAN, Switzerland) was applied to monitor the optical density (OD) at 450 nm after 4T1 cells were treated with NDs at diffrent concentrations.

### Hemolysis assay

The hemolysis reaction was employed to examine the blood compatibility of nanodroplets. The blood cells resuspended in PBS were added to different concentrations of NDs, incubated at 37℃ for 1 h, and the supernatant was added to a 96-well plate. The OD value of every well was determined at 545 nm by the microplate reader.

### In vivo biodistribution assessment

Upon the tumor volume reaching 200 mm^3^, Dil-marked HYD-NDs were injected intravenously into the mice. Subsequently, the mice underwent various time intervals of sacrifice (1, 2, 4, 8, and 24 h), during which tumors and primary organs were extracted. The study employed a Small Animal in vivo Imager (IVIS, PerkinElmer, Waltham, MA, USA) for both fluorescence imaging and quantification.

### Intracellular uptake

The ability of ultrasound to enhance cellular uptake of HYD-NDs was evaluated. 24-well plates were seeded with 4T1 cells overnight, and the cells were subsequently treated with media containing Dil-marked HYD-NDs for two hours with or without ultrasonic irradiation. The intracellular uptake of HYD-NDs was photographed by fluorescence microscope (Eclipse Ti2, Nikon, Tokyo, Japan).

The pH responsiveness of cells to HYD-NDs uptake was assessed. Using a fluorescent microscope, Dil-marked HYD-NDs were examined and photographed after being co-incubated with cells in a serum-free media at pH 7.4 and pH 6.5 for two hours.

### Liquid-gas phase transition

To detect the thermotropic phase transition, HYD-NDs was dropped on a glass slide inside the heating plate, the temperature of the heating plate was adjusted, and the phase transition of HYD-NDs was observed under an oil microscope.

To detect the acoustic phase transition, HYD-NDs were added into the well plate for ultrasonic irradiation (1.0 W/cm^2^, 30 s), and the irradiated HYD-NDs were dropped on a slide, and the phase transition was observed and compared under an oil microscope.

### Ultrasound imaging capability

The HYD-NDs solution was pipetted into the specially built examination model made of pipette drips for in vitro imaging. The model was then submerged in 37 °C aqueous solution and assessed using the ultrasound system (LOGIQ E9, GE, USA) with 9 L linear transducer to determine its ultrasound imaging capability.

Mice were anesthetized and injected with either 200 µL NDs, HYD-NDs or PBS via their vein for contrast enhanced ultrasound (CEUS) imaging in vivo. An ultrasound probe was then positioned over the tumor area and ultrasound imaging was performed according to the aforementioned parameters.

### LDH release assay

4T1 cells were planted and given several treatments in 96-well plates. Amounts of LDH seeping through injured cell membranes were used to assess the integrity of the cellular membrane, and then the OD value at 490 nm was recorded.

### Measurement of ROS

After 24 h of treatment in different ways, proceed according to the instructions to determine ROS using the fluorescent probe DCFH-DA. Photographs were taken by fluorescence microscope.

### Western blot

4T1 cells were subjected to various treatments, followed by cell harvesting and lysis using RIPA buffer supplemented with PMSF for 30 min. Protein samples from different experimental groups were used for subsequent experiments. The protein bands were analyzed by the chemiluminescence instrument (Tanon-4800, Shanghai, China).

### TEM imaging

Following a 24-hour period of varying treatments, 4T1 cells underwent digestion, were gathered, treated with 2.5% glutaraldehyde fixative. Following the fixative’s removal, 2% osmium tetroxide was used instead. The sections were then visualized using TEM.

### In vitro anti-tumor effect

4T1 cells were cultured with diverse treatments (PBS, NDs, Free HYD, HYD-NDs, NDs + US, HYD-NDs + US (1.0 W/cm^2^, 30 s, 1.0 MHz)), PBS served as control.

The CCK-8 assay was applied for assessing cell viability. Remove the old medium, add prepared CCK-8 solution and continue incubation for 1.5 h. Using a microplate reader, the OD values at 450 nm were determined.

The cell proliferation ability was evaluated utilizing the EdU-567 cell proliferation kit. The procedure was performed according to the kit procedure and then viewed under the fluorescence microscopy.

The transwell assay was applied to evaluate the capacity of cell invasion. 4T1 cells were treated differently and seeded at 4 × 10^4^ cells/well in a chamber containing matrigel, and the lower layer of the chamber was filled with media containing 15% FBS. Twenty-four hours after cultivation, the transell chambers were fixed, stained. Then viewed and photographed using a microscope.

### In vivo anti-tumor effect

Female BALB/c mice were utilized as an experimental model to observe the inhibitory effect of each treatment on tumor growth. Once the tumor reached a volume of 60 mm^3^, the mice were randomly divided into 6 groups (*n* = 5), the same group as in vitro. The dosage of HYD administered was 15 mg/kg, and ultrasound irradiation (1.5 W/cm^2^, 60 s, 1.0 MHz) was performed four hours after injection. The treatment duration lasted for twelve days during which the tumor volume and body weight of mice were recorded at two-day. At the end of observation period, all mice were sacrificed and tumor specimens were collected for HE staining, TUNEL staining, and immunohistochemical analysis (IHC). Furthermore, biological safety analyses were performed by means of tissue sections from primary organs.

### Statistical analysis

Every experiment has been performed independently at least three times. The statistical information was displayed as mean ± SD. Using GraphPad Prism 9 software, statistical analysis was carried out in accordance with the student’s t-test or one-way ANOVA. Statistics were considered significant when *p* < 0.05. *p < 0.05, ** *p* < 0.01, ***p < 0.001, ****p < 0.0001.

## Results and discussion

### Preparation and characterization of (HYD-)NDs

**Fig. 2:**
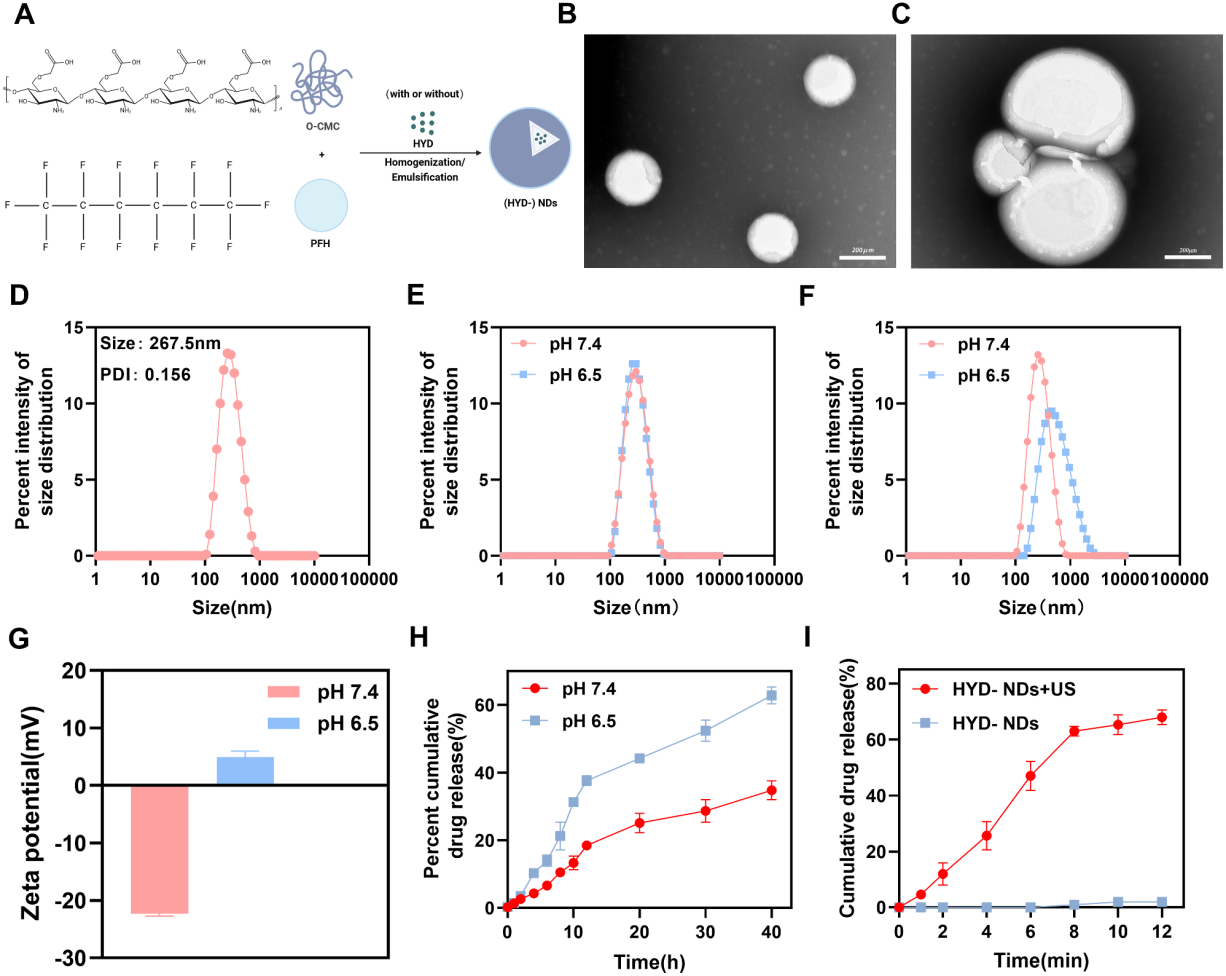
Characteristics of (HYD-)NDs. (**A**) (HYD)-NDs synthesis diagram. (**B**) HYD-NDs’ TEM scans under pH 7.4 conditions. (**C**) HYD-NDs’ TEM scans under pH 6.5 conditions. (**D**) The HYD-NDs’ size distribution under pH 7.4 conditions. (**E**) Comparison of the HYD-NDs size distribution over varied pH environments (0.5 h). (**F**) Size distribution comparison of HYD-NDs over diverse pH enviroulents (4 h). (**G**) Zeta potential of HYD-NDs over diffirent pH settings. (**H**) Drug release of HYD-NDs at varied pH settings. (**I**) HYD-NDs’ medication release profile when exposed to ultrasound. All data are provided as means ± SD (*n* = 3)

NDs and HYD-NDs were prepared using a homogeneous emulsification method based on a previously described protocol (Fig. 2A) [35, 36]. Direct observation using TEM showed that HYD-NDs had a unique core-shell structure and excellent dispersion at pH 7.4 (blood circulation’s acidity) (Fig. 2B), while swelling and aggregation occurred at pH 6.5 (the acidity of TME) (Fig. 2C). Further, DLS was used to measure the size variation of HYD-NDs. Under neutral conditions, HYD-NDs have the average size of 267.5 nm and the PDI of 0.156 (Fig. 2D). The size of HYD-NDs stayed nearly unaltered after incubation at pH 7.4 and pH 6.5 for 0.5 h (Fig. 2E), suggesting their stability in both blood circulation and TME for short period. They readily penetrated the endothelium of tumor tissue because of their small diameter and significant stability, and they accumulated locally as a result of increased permeability and retention (EPR) effects [37, 38]. Furthermore, the size of HYD-NDs was 268.62 nm and 472.47 nm respectively, after incubation for 4 h at pH 7.4 and pH 6.5 (Fig. 2F). Consistent with the TEM observations, there was no significant change in size in the neutral environment, but the particle size increased at pH 6.5.

Under acidic conditions, the zeta potential of HYD-NDs converted to positive values (4.93 ± 0.86 mV) while it remained negative under neutral conditions (-22.27 ± 0.37 mV) due to surface charge conversion properties (Fig. 2G). The negative charge on HYD-NDs’ surface prevents premature removal from blood circulation allowing it to circulate for longer periods while its charge conversion within tumor microenvironment promotes accumulation and cell internalization at tumor sites [14, 39]. Additionally, the particle size of HYD-NDs did not significantly change within 24 h when exposed to 50% FBS further confirming its stability during circulation (Fig. S1A).

The EE and LE of HYD in HYD-NDs were 71.84%±1.16% and 24.20%±0.30%, respectively (Fig. S1B). The pH and ultrasound responsiveness of the drug were evaluated by measuring drug release curves. Under neutral conditions, the 24-hour release rate of HYD was significantly lower than the cumulative release rate observed under acidic conditions (Fig. 2H). Furthermore, upon ultrasound irradiation, HYD exhibited an immediate release that the cumulative release rate of 63.0% within 8 min before transitioning into a sustained release profile (Fig. 2I). These findings indicate that HYD-NDs can achieve targeted release at the tumor site under the dual stimulation of pH and ultrasound, thereby minimizing drug release from normal tissues and reducing potential side effects.

### Biosafety and tumor targeting ability

**Fig. 3:**
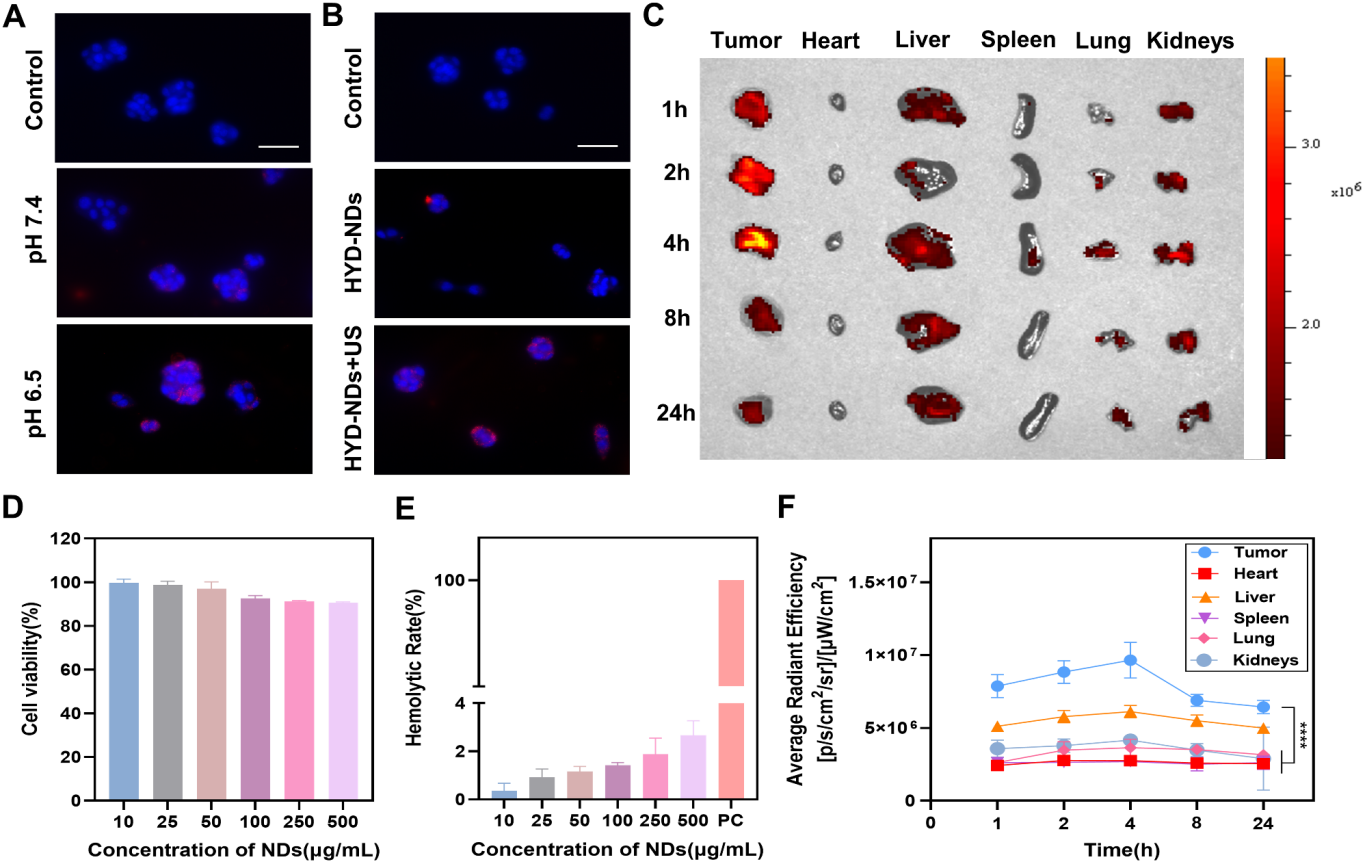
Biocompatibility, biodistribution and intracellular uptake. (**A**) Cellular uptake of Dil-marked HYD-NDs under varied pH settings. Scale bar: 100 μm. (**B**) Cellular uptake of HYD-NDs marked with Dil following ultrasonic irradiation. Scale bar: 100 μm. (**C**) Fluorescence images captured of isolated tumors and main organs at different time intervals. (**D**) NDs’ in vitro biocompatibility at different doses for a 24-hour treatment period. (**E**) In vitro hemolysis assay with different doses of NDs, using deionized water as a positive control. (**F**) The Dil-marked HYD-NDs accumulation curve in tumors and major organs was produced using Fig. C. All data are provided as means ± SD (*n* = 3)

To assess the biocompatibility of NDs, initial cytotoxicity experiments demonstrated that the viability of 4T1 cells remained above 90% even after co-incubation with varying concentrations of NDs for 24 h (Fig. 3D). Furthermore, hemolysis test results indicated that even at a concentration as high as 500 ug/mL, the hemolysis rate was still below 5% (Fig. 3E). The results confirmed that the designed NDs had good biocompatibility and was suitable for use as the drug delivery system.

Passive phagocytosis and ultrasound-mediated cavitation were identified as mechanisms through which cells internalize NDs [40]. The internalized cellular uptake of HYD-NDs was considerably increased in an acidic environment (the tumor microenvironment) or in following ultrasonic irradiation, as shown in Fig. 3A and B. The primary causes of this increased uptake are the sonoporation produced on by ultrasound and the charge conversion of HYD-NDs within TME, which stimulates the electrostatic attraction to cancer cells [41–43].

Additionally, biodistribution analysis using Dil-marked HYD-NDs further investigated their distribution in tumors and major organs. The fluorescence intensity in the tumor site reached its maximum at four hours following injection (Fig. 3C and F). Enhanced Dil aggregation at tumor site could be attributed to EPR effect and charge conversion within tumor microenvironment facilitated by HYD-NDs [44]. Notably, retention time of HYD-NDs in tumor area extended beyond 24 h, and it is suggested that NDs can achieve long-term stable accumulation in tumors, which is more conducive to the effect of loaded drugs. Alternatively, accumulation in liver was observed due to phagocytosis by reticuloendothelial system [45].

### Liquid-gas phase transition

PFH, the core of the HYD-NDs, has proven to be a desirable phase transition material that can be triggered by a variety of factors (ultrasound, temperature, laser, etc.) [46]. We verified the effects of temperature and ultrasound on the liquid-gas phase transition of HYD-NDs. As shown in the Fig. S2A, the volume of nanodroplets increased significantly after ultrasound irradiation compared with Control group, indicating that ultrasound can induce liquid-air phase transition of HYD-NDs. While no significant changes were observed in the nanodroplets at 25 °C and 37 °C (Fig. S2B), indicating that the nanodroplets can remain stable at room and physiological temperatures, which also ensures that they will not be induced to undergo a phase transition due to the surrounding ambient temperatures when applied in vitro and in vivo. Furthermore, we also detected the size of HYD-NDs after ultrasound by dynamic light scattering (Fig. S2C), and the results showed that the average size of HYD-NDs increased significantly after ultrasound, which also proved that it underwent a phase transition.

### Ultrasound imaging capability

**Fig. 4:**
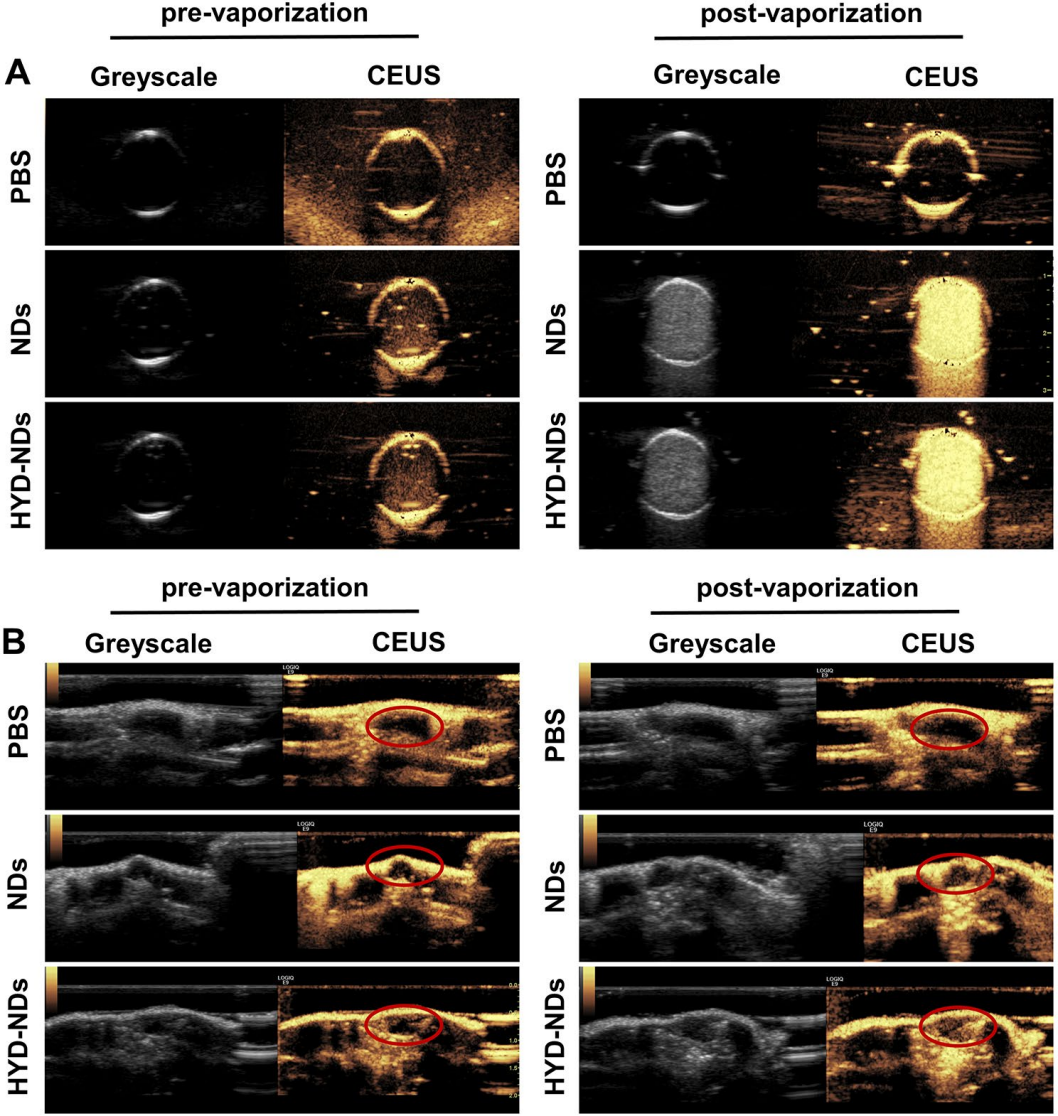
CEUS of NDs and HYD-NDs. (**A**) Ultrasound imaging in vitro. (**B**) Ultrasound imaging in vivo. Tumor site shown by red circle

To evaluate the imaging capability, ultrasound imaging was obtained both in vivo and in vitro using grayscale and CEUS modes. When compared to PBS, after ultrasound irradiation, NDs and HYD-NDs showed superior echogenicity in CEUS mode in vitro (Fig. 4A and S3A). This may be the result of the ADV of the PFH-core nanodroplets to form microbubbles [26]. In vivo, the tumor was almost devoid of echogenic signal following intravenous injection of PBS, in contrast, the NDs and HYD-NDs groups displayed a significant increase in echo after ultrasound irradiation, allowing for clear visualization of the tumor margin and size (Fig. 4B and S4B). Furthermore, Fig. S3 also showed that there were no significantly difference between the NDs and HYD-NDs groups both before and after the phase transition. According to the data above, NDs are a great ultrasound contrast agent that may be utilized to achieve an integrated diagnosis and treatment mode, increase treatment precision and controllability.

### Mechanism of UTMD combined with HYD-NDs inducing pyroptosis

**Fig. 5:**
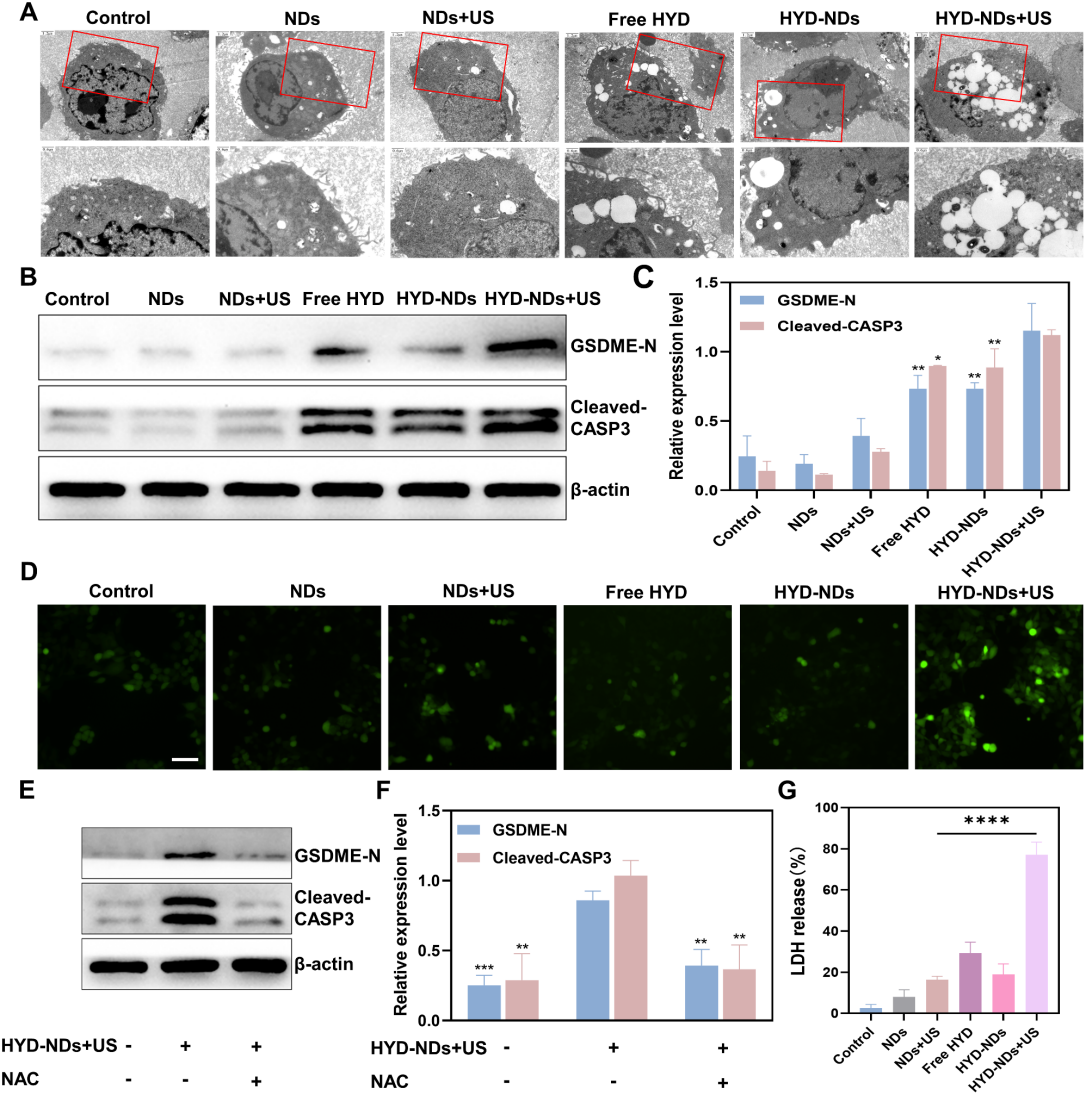
Mechanism of UTMD in combination with HYD-NDs inducing pyroptosis. (**A**) TEM photographs of 4T1 cells with different treatments. The red boxes in the upper image can be seen in greater detail in the bottom figures (magnification 2X). Scale bar: 1.2 μm, 0.6 μm. (**B**) GSDME-N and Cleaved-CASP3 relative expression levels on tumor cells treated differently. (**C**) Quantification of GSDME-N and Cleaved-CASP3 was performed from B. (**D**) Fluorescence photographs showing the production of ROS in 4T1 cells following various treatments. Scale bar: 100 μm. (**E, F**) The expression level (E) and quantitative analysis (**F**) of GSDME-N and Cleaved-CASP3 in 4T1 cells following different therapies. (**G**) Release of LDH in culture supernatants. All data are provided as means ± SD (*n* = 3)

The process of pyroptosis induced by HYD-NDs combined with UTMD was observed using TEM (Fig. 5A). The cells treated with HYD-NDs combined with US exhibited characteristic indications of pyroptosis, that includes membrane pore creation and membrane leakage, in comparison to the other groups. To verify the leaking of cell contents, LDH release from the cell cultured supernatant was further measured [47]. As shown (Fig. 5G), the amount of LDH released from the supernatant of cells treated with HYD-NDs with US had greatly higher than that of cells treated with Free HYD and HYD-NDs (*p* < 0.0001). This indicated that combined treatment in 4T1 cells obviously induced pyroptosis.

Next, the levels of proteins related to pyroptosis in 4T1 cells were detected by Western blotting to further explore the mechanism of pyroptosis. GSDME is a specific pyroptosis substrate for caspase-3 cleavage [48]. As observed in the figures (Fig. 5B and C), compared with the HYD or HYD-NDs groups, the HYD-NDs + US treatment group had a more significant increase in GSDME-N (*p* < 0.01). Corresponding to this result was an increase in caspase-3 lyase. Semi-quantitative results confirmed that HYD-NDs combined with US could activate caspase-3 to cleave GSDME, thereby achieving pyroptosis, which was significantly better than Free HYD or HYD-NDS alone.

Furthermore, treatment with HYD-NDs + US led to a notable rise in ROS, shown by DCFH-DA’s fluorescence (Fig. 5D). Notably, treatment with ROS inhibitor NAC significantly attenuated the changes in pyroptosis morphology, GSDME cleavage and caspase-3 activation (Fig. 5E and F), indicating that ROS removal effectively prevented pyroptosis induced by HYD-NDs + US. These results are consistent with previous fingdings that indicated ROS is upstream of the caspase3/GSDME signaling pathway [33, 34]. The findings showed that, following HYD-NDs with UTMD therapy, ROS was the main cause of pyroptosis in 4T1 breast cancer cells.

Taken together, the above results demonstrate that HYD-NDs combined with UTMD can initiate the pyroptosis process and that the combined strategy could be an effective way to optimize the therapeutic impact of cancer treatment.

### In vitro anti-tumor effect

**Fig. 6:**
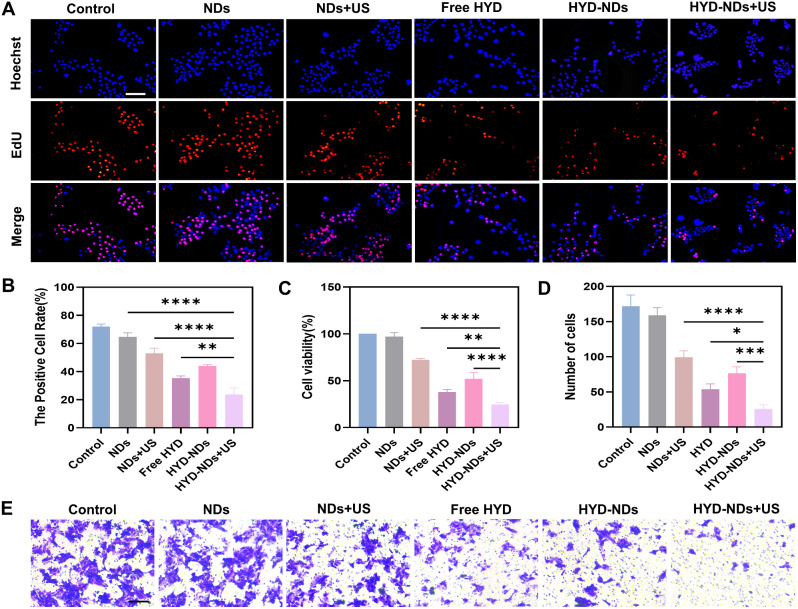
In vitro anti-tumor efficiency assessment. (**A**) Fluorescence photographs showing the ability of cells to proliferate under different treatments for a 24-hour period. Scale bar:100 μm. (**B**) The proportion of positive cells was extracted from A. (**C**) Cell viability with different treatments. (**D**) Quantification of the invading cells in Fig. E. (**E**) Invasion experiment for 24 h with various treatments. Scale bar: 100 μm. The means ± SD (*n* = 3) are given for all data analysis

The CCK-8 assay was applied to asses cell viability. The findings displayed that, in comparison to the other groups, the HYD-NDs + US group had the lowest cell viability (24.81%±1.26%) (Fig. 6C), indicating that the combination therapy’s cytotoxic effect was more effective than the monotherapy’s.

The anti-proliferation effect of various therapies on 4T1 cells was measured using the EdU kit. The results were consistent with the pattern shown in the CCK-8 assay, and the HYD-NDs + US group had the least percentage of positive cells (Fig. 6A and B). This indicates that the combination treatment could effectively inhibit the proliferation of 4T1 cells.

Additionally, the capacity of cells to invade was determined using the transwell assay. The findings showed that the HYD-NDs + US group’s invasion rate was 23.86 ± 3.81%, considerably lower than the other groups’ rates (Fig. 6D and E).

Taken together, the findings above suggest that the combination treatment greatly decreases cell survival and inhibits cell invasion and proliferation through a synergistic impact.

### In vivo anti-tumor effect

**Fig. 7:**
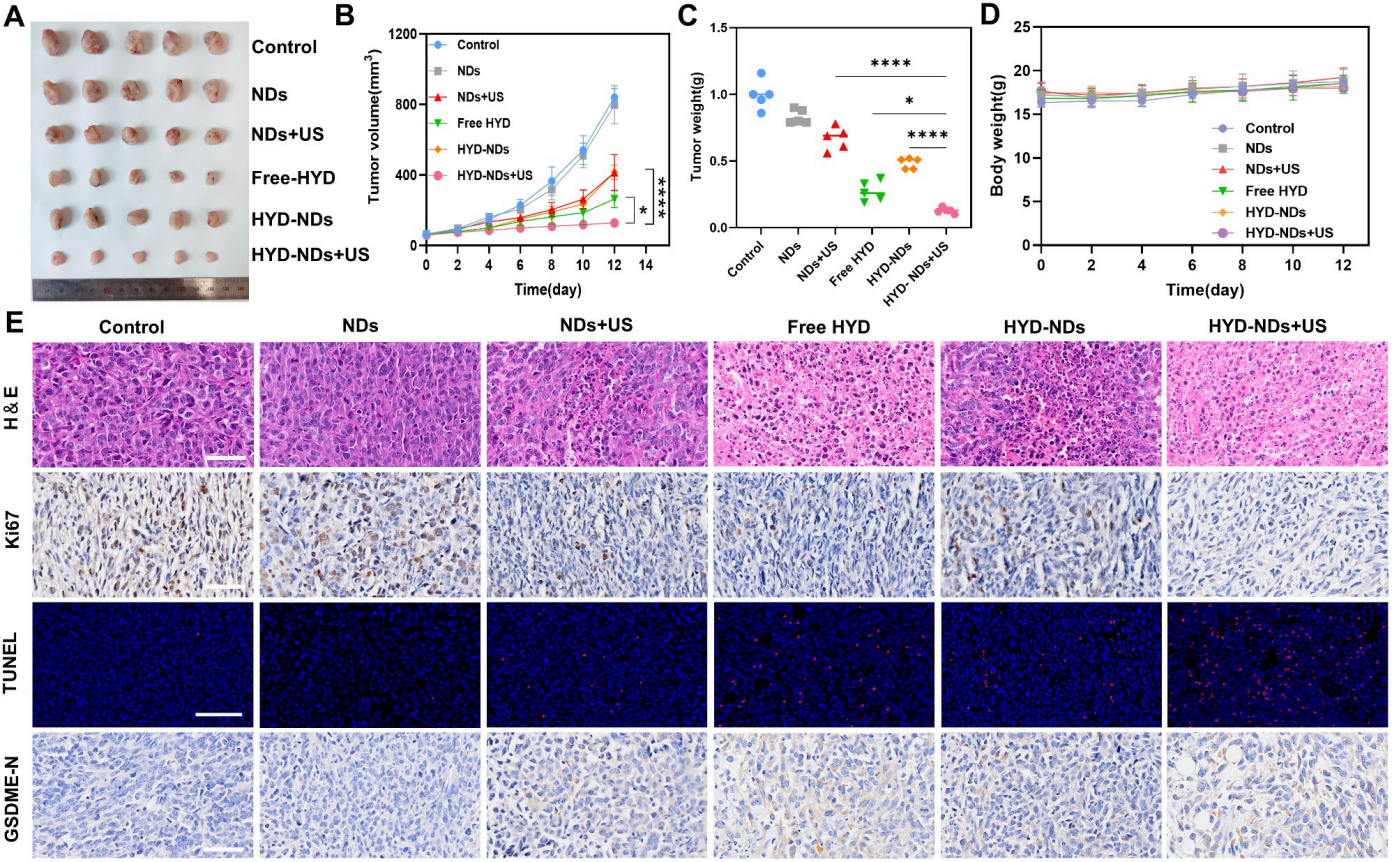
In vivo anti-tumor effect. (**A**) Pictures of the collected tumors following various treatments. (**B**) Tumor volume change curves following various treatments. (**C**) Weight of ex vivo tumors in the groups. (**D**) Weight change curves for mice following various treatments. (**E**) IHC, TUNEL, and HE staining of tumors treated with various strategies. Scale bar:100 μm. The means ± SD (*n* = 3) are given for all data analysis

Further evaluation of the antitumor effects of HYD-NDs combined with UTMD in a 4T1 breast cancer xenograft mouse model. Mice were sacrificed at the ending of the trial, and tumors were measured and photographed. As shown (Fig. 7A, B and C), the NDs group showed nearly no inhibitory impact when compared to the control group. Furthermore, we discovered that the tumor-inhibitory effect of the free HYD group was greater than that of the HYD-NDs group. This was mostly because the nanodroplets could release gradually without being stimulated by ultrasound. The tumor growth inhibition (TGI) of the HYD-NDs + US group was 87.15%, and its tumor growth rate was considerably lower than that of the other groups. The HYD-NDs + US group’s tumor weight (0.13 ± 0.02 g) was 3.69 times heavier than that of the HYD-NDs group (0.48 ± 0.04 g) and 5.15 times heavier than that of the NDs + US group (0.67 ± 0.08 g), proving the full superiority of HYD-NDs in combination with UTMD. The findings of TUNEL, IHC, and HE staining were consistent with the results shown above (Fig. 7E). The IHC labeling of Ki67, an indication of cell proliferation, revealed that the cell proliferation was dramatically lowered in the HYD-NDs combined with UTMD treatment group. The HYD-NDs + US group also showed enhanced apoptosis and apparent nuclear shrinkage and fragmentation. All of these results confirmed the combination of therapy’s remarkable anticancer effect.

Furthermore, GSDME was stained with IHC to assess its expression in the tumor tissue. The expression of GSDME in the HYD-NDs + US group was considerably greater than that of both the HYD and NDs + US groups, which was in line with the in vitro results (Fig. 7E).

Each group of mice had a 100% survival rate at the end of the experiment. As shown, there was no discernible variation in the groups’ body weights over the period of the treatment (Fig. 7D) (*p* > 0.05). Furthermore, the major organs stained with HE in each group did not exhibit pathological alterations (Fig. S4), indicating the good biosafety and biocompatibility of HYD-NDs in vivo.

## Conclusion

In this study, we successfully constructed HYD-loaded nanodroplets with PFH as core and O-CMC as coating material. HYD-NDs offer both endogenous acid-responsive and exogenous US-responsive drug fast-release properties, as well as favorable imaging properties and biosafety. Furthermore, by preventing the methylation of the DNA promoter in tumor cells, HYD-NDs can enhance the expression of GSDME. On the other hand, HYD-NDs combined with UTMD treatment can increase the level of ROS in cells, activate caspase-3, and cleave GSDME to cause pyroptosis. Experiments conducted both in vitro and in vivo confirmed the good anti-tumor effect of HYD-NDs in combination with UTMD. Our results may provide a prescription for the treatment of tumors with GSDME gene silencing and have the potential to guide the development of clinical treatment modalities.

### Electronic supplementary material

Below is the link to the electronic supplementary material.

Supplementary Material 1

## Data Availability

Data from this study are available upon request from the corresponding author.
