# Supplementary material for: Hydralazine loaded nanodroplets combined with ultrasound-targeted microbubble destruction to induce pyroptosis for tumor treatment
Source: J Nanobiotechnology. 2024 Apr 20;22:193. doi: 10.1186/s12951-024-02453-0 (PMC11031971; doi:10.1186/s12951-024-02453-0)
Supplement: Supplementary file 1 — Supplementary Material 1 [file 12951_2024_2453_MOESM1_ESM.docx]

**Supporting Information**

**Hydralazine loaded nanodroplets combined with ultrasound-targeted microbubble destruction to induce pyroptosis for tumor treatment**

Shuting Huang^a^, Mengmeng Shang^a^, Lu Guo^a^, Xiao Sun^a^, Shan Xiao^a^, Dandan Shi^a^, Dong Meng^a^, Yading Zhao^a^, Xiaoxuan Wang^a^, Rui Liu^a^, Jie Li ^a,b,*^

^a^ Department of Ultrasound, Qilu Hospital of Shandong University, Jinan, Shandong 250012, China

^b^ Department of Ultrasound, Qilu Hospital (Qingdao) of Shandong University, Qingdao, Shandong 266035, China

^*^Corresponding authors: Tel/fax: +86-531-82166101. E-mail address: jieli@email.sdu.edu.cn

This part included:

Figure S1-4


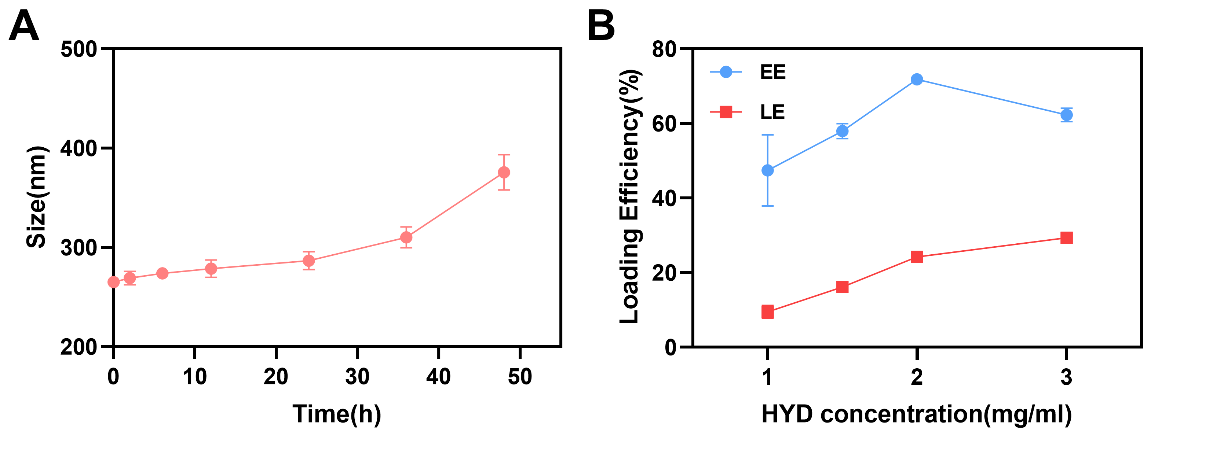


**Fig.S1** Characteristics of HYD-NDs. (A) The size change of HYD-NDs in 50% FBS for 48 h.(B) EE and LE of HYD-NDs. Data are presented as means ± SD (n = 3).


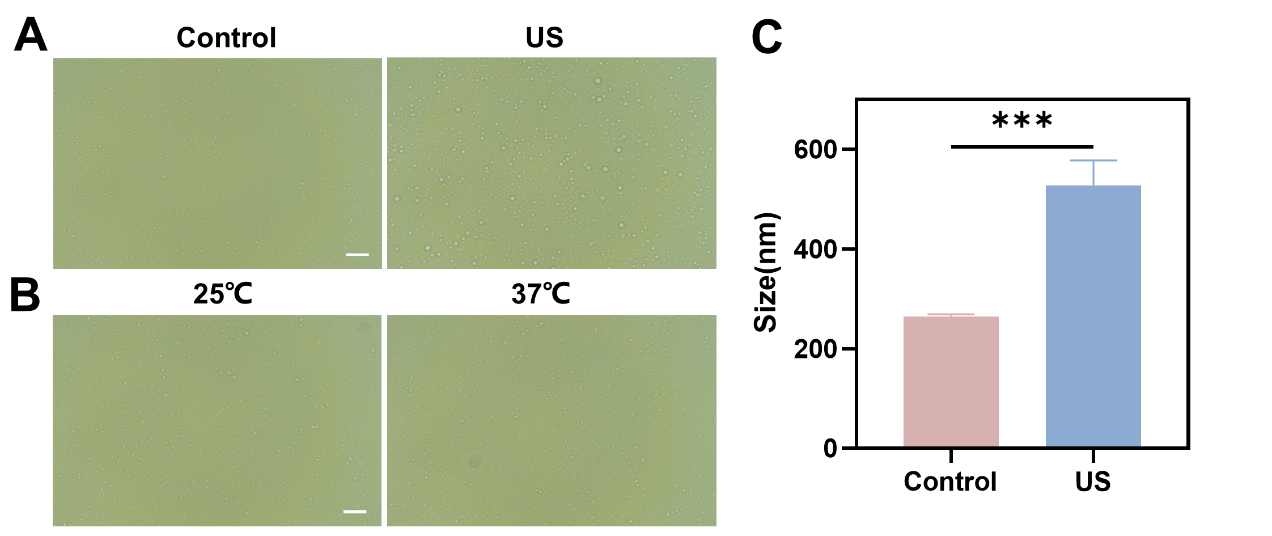


**Fig.S2** Photomicrographs of HYD-NDs undergoing liquid-gas phase transition. (A). Volume change of HYD-NDs under ultrasonic irradiation. Scale bar: 10 μm. (B). Volume change of HYD-NDs at different temperatures. Scale bar: 10 μm. (C). The size of HYD-NDs after ultrasound irradiation. ****p* < 0.001.


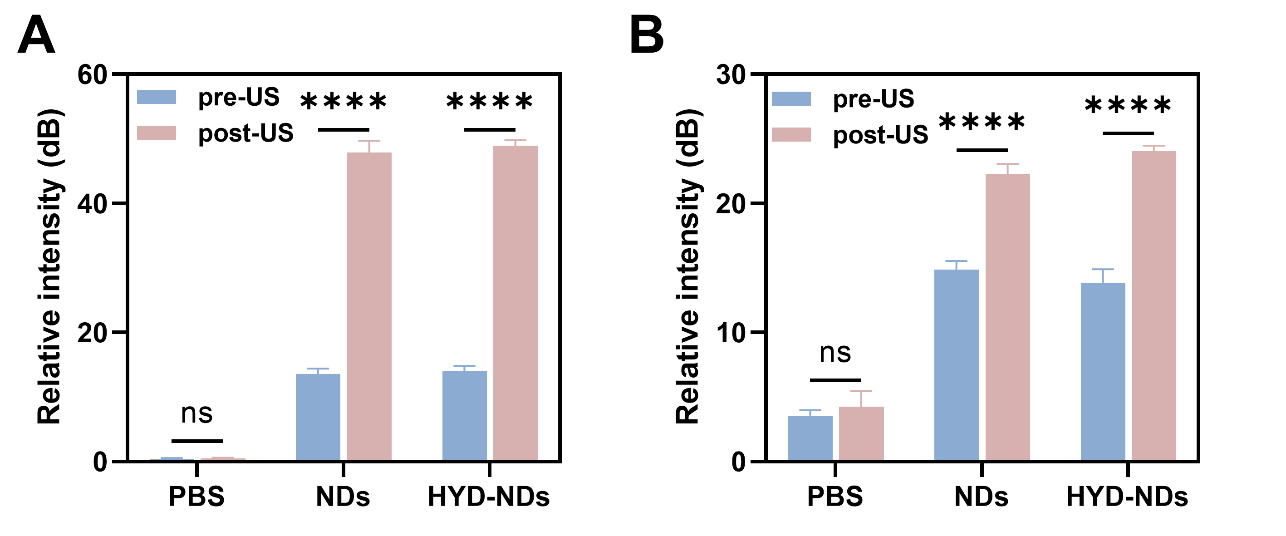


**Fig.S3 (**A). Quantification of ultrasound signal intensity from A. (B). Quantification of ultrasound signal intensity from B. *****p* < 0.0001.


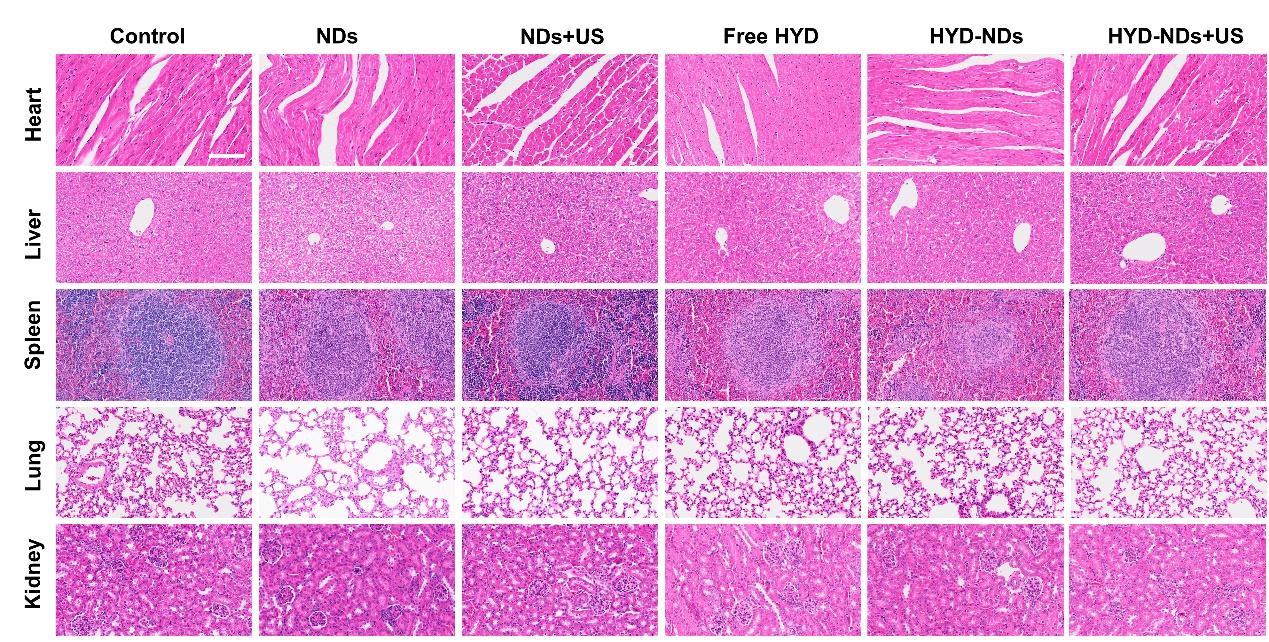


**Fig.S4** HE staining of major organs in the different groups. Scale bar: 100 μm.
